# An Enigmatic Case of Rectal Bleeding in a Young Woman—A Forgotten Intrauterine Device, Perforation and Rectal Involvement: A Case Report and Literature Review

**DOI:** 10.3390/jcm15145637

**Published:** 2026-07-18

**Authors:** Libby Or Madar, Ariel Polonsky, Ilan Bruchim, Oren Gal

**Affiliations:** 1 Department of Obstetrics and Gynecology, Hillel Yaffe Medical Center, Hadera 3820302, Israel; libbymdr@gmail.com (L.O.M.); ilan.bruchim@gmail.com (I.B.); 2 Ruth and Bruce Rappaport Faculty of Medicine, The Technion—Israel Institute of Technology, Haifa 3200003, Israel; 3 Department of Gastroenterology and Hepatology, Hillel Yaffe Medical Center, Hadera 3820302, Israel

**Keywords:** IUD perforation, multidisciplinary team, laparoscopy

## Abstract

**Background**: Perforation associated with intrauterine devices (IUDs) occurs in approximately 1 in 1000 insertions and may be either partial or complete. Perforation may be primary, occurring during device insertion, or secondary, developing after the device has remained in situ for more than eight weeks. Typical symptoms of IUD perforation include chronic pain and intestinal obstruction. Rarely, the device is found either completely within the rectal lumen or partially embedded in the rectal wall with partial intraluminal extension. Removal of the device may require colonoscopy, laparoscopy, or a combination of both. Case: A 45-year-old woman was referred for evaluation of intermittent rectal bleeding. Initial outpatient evaluation included contrast-enhanced computed tomography (CT), which demonstrated two intrauterine devices: one appropriately positioned within the uterine cavity and another located within the rectouterine pouch. A multidisciplinary discussion involving gastroenterologists, gynecologists, and colorectal surgeons was subsequently conducted. Under general anesthesia, laparoscopy was initiated. Upon entering the abdominal cavity, a free IUD string was visualized embedded within the pelvic peritoneum. Using simultaneous colonoscopic guidance with transillumination, a targeted peritoneal incision was made overlying the IUD. The arms were removed laparoscopically. Subsequent colonoscopy removed the remaining segment of the IUD traversing the rectal wall. **Conclusions**: Although uterine perforation and migration of intrauterine devices are uncommon, they may result in severe and potentially life-threatening complications. Early diagnosis, careful documentation, routine follow-up, and timely removal of misplaced devices remain essential to minimizing morbidity and preventing adverse outcomes.

## 1. Introduction

Perforation associated with intrauterine devices (IUDs) occurs in approximately 1 in 1000 insertions and may be classified as either partial or complete [1]. In cases of complete perforation, the device may migrate into the pelvic or abdominal cavity, invading adjacent organs in nearly 15% of cases [1,2]. Although uncommon, migration may lead to serious complications involving the bowel, bladder, omentum, or peritoneal cavity, occasionally presenting years after insertion.

Perforation may be primary, occurring during insertion, or secondary, developing after prolonged residence of the device within the uterus. Primary perforation is associated with factors such as operator inexperience, abnormal uterine position, postpartum status, lactation, and uterine atrophy [2,3]. Secondary perforation is thought to result from gradual myometrial erosion caused by chronic uterine contractions and local inflammatory reactions, allowing migration into adjacent pelvic organs, including the rectum, sigmoid colon, bladder, or small intestine [4,5].

Clinical presentation ranges from asymptomatic cases to chronic pelvic pain, abnormal bleeding, bowel obstruction, or pelvic infection [4]. Rectal involvement is particularly rare but may present with rectal bleeding, perforation, fistula formation, or pelvic sepsis [4,6]. While endoscopic retrieval is often appropriate when the device lies predominantly within the rectal lumen, laparoscopic or combined laparoscopic–endoscopic removal is generally preferred when a substantial portion of the IUD is embedded within the rectal wall, allowing safe extraction and immediate repair if necessary [2].

We report a rare case of uterine perforation detected approximately twenty years after IUD insertion. The migrated device partially penetrated the rectal wall and presented with intermittent rectal bleeding. The patient was successfully treated using a combined laparoscopic and colonoscopic approach.

### 1.1. Case Description

A 45-year-old woman was referred for evaluation of intermittent rectal bleeding. Initial outpatient evaluation included contrast-enhanced computed tomography (CT), which demonstrated two intrauterine devices: one appropriately positioned within the uterine cavity and another located within the rectouterine pouch. A review of her medical records revealed that an IUD had been inserted approximately twenty years earlier, not during her postpartum period. Thirteen years later during a routine Gyn examination, the strings of the IUD were not visualized, and as the device was not observed on US inside the uterus, it was assumed by her Gyn that it was spontaneously expelled. As the patient wished for a new IUD, a device was inserted into the uterus successfully.

**Figure 1:**
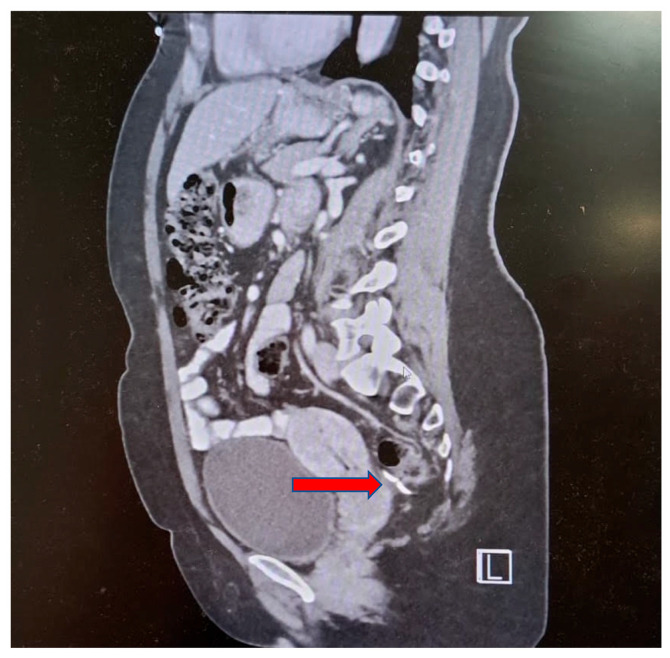
Sagittal image of an abdominal CT scan showing the IUD arms embedded in the rectal wall.

Further workup included outpatient clinic gastroscopy and colonoscopy, both of which were initially reported as adequate and normal. Owing to the unusual CT findings (Figure 1), the patient was referred for gynecologic evaluation.

**Figure 2:**
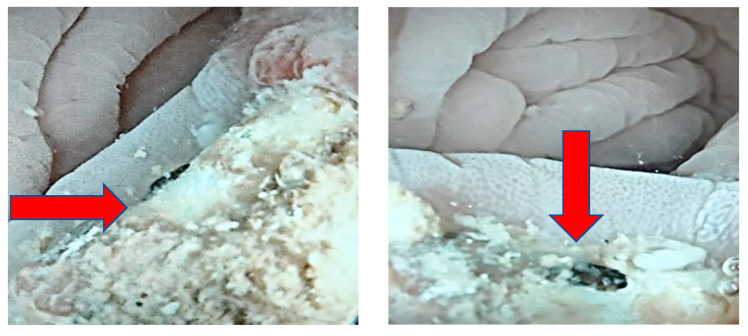
Endoscopic views of the rectum demonstrating normal rectal folds in the background and, in the distal field, debris surrounding a small dark foreign body. The appearance is consistent with intraluminal visualization of a perforated intrauterine device (IUD) that migrated into the rectal lumen.

Pelvic ultrasonography (US) suggested that the arms of the extrauterine IUD were positioned posterior to the cervix and adjacent to the rectum, raising suspicion for rectal wall involvement. Given these findings, repeat colonoscopic evaluation was performed at our institution. During this examination, a foreign body resembling an IUD was identified approximately 15 cm from the anal verge, corresponding anatomically to the rectosigmoid junction (Figure 2).

A multidisciplinary discussion involving gastroenterologists, gynecologists, and colorectal surgeons was subsequently conducted. Nevertheless, due to the likelihood that the device had remained embedded for many years and the associated risks of perforation and infection, elective operative removal was recommended.

After thorough discussion of management options, the team elected to proceed with a combined laparoscopic and colonoscopic approach in the operating room. The aim was to maximize visualization, minimize bowel injury, and allow immediate repair if necessary.

Under general anesthesia, laparoscopy was initiated. Upon entering the abdominal cavity, a free IUD string was visualized embedded within the pelvic peritoneum. The arms of the device were identified beneath the peritoneum along the left anterior rectal wall. Notably, there were no significant pelvic adhesions, abscesses, or intra-abdominal fluid collections.

Using simultaneous colonoscopic guidance with transillumination, a targeted peritoneal incision was made overlying the IUD about 15 cm above the anal verge. Careful dissection of dense fibrotic tissue permitted exposure of the embedded arms of the device. Gentle traction demonstrated that the arms had detached from the vertical stem of the IUD. The detached arms were removed laparoscopically without difficulty.

Subsequent colonoscopy revealed the remaining segment of the IUD traversing the full thickness of the rectal wall, with portions protruding into the lumen. The retained fragment was successfully extracted endoscopically. Following removal, the rectal mucosa was approximated using three endoscopic clips, while the muscularis layer was reinforced laparoscopically using an Endo GIA stapling device. Leak testing with diluted methylene blue demonstrated no evidence of extravasation. The pelvic peritoneum was then closed with a V-Loc suture. Finally, the second intrauterine device was removed transvaginally. No additional intervention was used postoperatively.

The patient’s postoperative course was uneventful. She was discharged three days later.

### 1.2. Discussion and Review of the Literature

IUD perforation is generally classified as either partial or complete, depending on the depth of penetration through the uterine wall [2]. Partial perforation involves embedding within the myometrium, whereas complete perforation refers to total migration through the serosa into the peritoneal cavity or adjacent organs [7]. Although uncommon, complete perforation may result in serious complications requiring surgical intervention.

The World Health Organization recommends prompt removal of all extrauterine IUDs regardless of symptomatology, due to the risk of adhesions, fistula formation, bowel perforation, and pelvic infection [8]. This recommendation is particularly important because migrated devices may remain asymptomatic for prolonged periods, delaying diagnosis until severe complications arise.

Our case highlights several important clinical considerations. First, IUD migration may remain undetected for decades. Second, symptoms may be nonspecific and unrelated to gynecologic complaints. Finally, multidisciplinary collaboration is essential when involvement of adjacent organs is suspected.

Several reports in the literature describe severe complications resulting from migrated IUDs. In 2009, a case series described four women presenting with intraperitoneal sepsis and complex pelvic masses caused by perforated copper IUDs. Surgical management in all cases was complicated by extensive adhesions surrounding the devices [9].

Another report described a 59-year-old woman with persistent vaginal discharge and chronic pelvic pain. Initial US was unrevealing, and repeated antibiotic therapy failed to improve symptoms. CT imaging eventually demonstrated a fistula between the uterus and small bowel. During surgery, the IUD was identified adjacent to the fistulous tract, necessitating hysterectomy and bowel resection [10].

Rectal perforation secondary to IUD migration represents one of the most severe gastrointestinal complications [4,6]. Günakan et al. described a 33-year-old woman who presented three years after IUD insertion with fever, abdominal pain, and vaginal discharge. Pelvic MRI demonstrated an extrauterine IUD associated with bilateral pelvic abscesses. Exploratory laparotomy revealed a frozen pelvis and rectosigmoid perforation caused by device migration. Management required subtotal hysterectomy, salpingo-oophorectomy, and loop colostomy [11].

Another notable case was described by Bello et al. involving a 31-year-old woman who became pregnant despite IUD placement. Following delivery, she later noticed the IUD strings protruding through her anus. Imaging confirmed migration of the device posterior to the uterus and penetration through the rectal wall. Under anesthesia, the device was removed transanally without complication [12].

Additional reports describe asymptomatic patients in whom migrated devices were discovered incidentally during imaging or follow-up examinations. In one such case, a 37-year-old woman underwent evaluation after missing IUD strings were noted. Initial laparoscopy failed to identify the device; however, CT imaging later demonstrated migration into the lower rectum. Sigmoidoscopic retrieval was performed successfully without evidence of residual perforation [13].

A comprehensive literature review identified only 15 reported cases of rectal perforation associated with IUD migration, emphasizing the rarity of this complication [14]. Interestingly, five of these cases occurred within six months of delivery, supporting the hypothesis that postpartum uterine vulnerability contributes significantly to the risk of perforation. Most reported cases were ultimately managed successfully using endoscopic techniques [14].

Our case contributes to the growing body of literature demonstrating that minimally invasive combined approaches can provide safe and effective treatment for complex migrated IUDs involving the bowel. Simultaneous laparoscopy and colonoscopy allowed accurate localization, controlled dissection, immediate repair of the rectal defect, and avoidance of major bowel resection.

In conclusion, although uterine perforation and migration of intrauterine devices are uncommon, they may result in severe and potentially life-threatening complications. Clinicians should maintain a high index of suspicion in patients with missing IUD strings, chronic pelvic symptoms, unexplained gastrointestinal complaints, or abnormal imaging findings. Early diagnosis, careful documentation, routine follow-up, and timely removal of misplaced devices remain essential for minimizing morbidity and preventing adverse outcomes.

Teaching points:

1. Failure to visualize an IUD on US does not necessarily indicate spontaneous expulsion.
2. A migrated IUD may result in serious complications and should be promptly localized and removed.
3. Management of a rectally embedded IUD is complex and should be carefully planned by a multidisciplinary team.

## Data Availability

This is a descriptive case report. No research data was published here, so there are no relevant data to share.

## References

[1] Sitruk-Ware R., Nath A., Mishell D.R. (2013). Contraception technology: Past, present and future. Contraception.

[2] Rowlands S., Oloto E., Horwell D.H. (2016). Intrauterine devices and risk of uterine perforation: Current perspectives. Open Access J. Contracept..

[3] Zhou X., Ichikawa L., Armstrong M.A., Raine-Bennett T., Getahun D., Asiimwe A., Pisa F., Schoendorf J., Reed S.D., Gatz J.L. (2022). Intrauterine device-related uterine perforation incidence and risk (APEX-IUD): A large multisite cohort study. Lancet.

[4] Kaislasuo J., Suhonen S., Gissler M., Lähteenmäki P., Heikinheimo O. (2013). Uterine perforation caused by intrauterine devices: Clinical course and treatment. Hum. Reprod..

[5] Goldstuck N.D., Wildemeersch D. (2014). Role of uterine forces in intrauterine device embedment, perforation, and expulsion. Int. J. Womens Health.

[6] Buhling K.J., Zite N.B., Lotke P., Black K. (2014). Worldwide use of intrauterine contraception: A review. Contraception.

[7] Aliukonis, Lasinskas M., Pilvelis A., Gradauskas A. (2020). Intrauterine device migration into the lumen of large bowel: A case report. Int. J. Surg. Case Rep..

[8] WHO Scientific Group on Mechanism of Action, Efficacy of Intrauterine Devices (1987). Mechanism of Action Safety and Efficacy of Intrauterine Devices: Report of a WHO Scientific Group.

[9] Pillai M., Van de Venne M., Shefras J. (2009). Serious morbidity with long-term IUD retention. J. Fam. Plan. Reprod. Health Care.

[10] Desai A.Y., Dnyaneshwar S., Pai V.D. (2015). Forgotten Intrauterine Contraceptive Device—An Unusual Cause of Enterouterine Fistula. J. Surg..

[11] Günakan E., Buluş H., Polat F. (2018). Colonic perforation due to the migration of an intrauterine device (IUD): Surgical manage-ment for acute abdomen. Ortadoğu Tıp Derg..

[12] Bello O.O., Ayandipo O.O., Olayide R.O. (2018). Case report: Retrieval of an intra-uterine contraceptive device penetrating through the wall of the rectum. Ann. Ib. Postgrad. Med..

[13] Ma G.W., Yuen A., Vlachou P.A., de Montbrun S. (2016). An unconventional therapeutic approach to a migratory IUD causing perforation of the rectum. J. Surg. Case Rep..

[14] Boushehry R., Al-Taweel T., Bandar A., Hasan M., Atnuos M., Alkhamis A. (2022). Rare case of rectal perforation by an intrauterine device: Case report and re-view of the literature. Int. J. Surg. Case Rep..

